# October 7th 2023 mass casualty incident in southern Israel: lessons for emergency preparedness and management

**DOI:** 10.1186/s13584-024-00651-7

**Published:** 2024-11-11

**Authors:** Aya Gozlan, Ran Abuhasira, Jacob Dreiher, Shosh Peleg, Gilbert Sebbag, Dan Schwarzfuchs, Tzachi Slutsky, Dror Dolfin, Amit Frenkel, Shlomi Codish

**Affiliations:** 1 Hospital Administration, Soroka University Medical Center, Beer Sheva, Israel; 2 Hospital Administration and Emergency Department, Soroka University Medical Center, Beer Sheva, Israel; 3 Intensive Care Unit, Soroka University Medical Center, Beer Sheva, Israel; 4 Surgery Division, Soroka University Medical Center, Beer Sheva, Israel; 5 Clinical Research Center, Soroka University Medical Center, Beer Sheva, Israel; 6 Hospital Medical Management, Soroka University Medical Center and Faculty of Health Sciences, Ben-Gurion University of the Negev, Be’er-Sheva, Israel

## Abstract

**Background:**

Mass Casualty Incidents (MCIs) pose significant challenges for healthcare systems. While policies are typically crafted based on past experiences, the lessons learned from each incident play a crucial role in enhancing emergency preparedness.

On October 7th, 2023, Israel came under the largest terror attack in its history. During an ongoing terror attack, more than 1300 Israelis were killed, and more than 200 were abducted to Gaza. During the first day of the attack, 1457 casualties were evacuated to a hospital, approximately half of them to Soroka University Medical Center (SUMC). This MCI surpassed conventional MCI challenges, necessitating a need to face the unexpected under fire.

Through a description of this extreme MCI, we delve into the challenges faced, the strategic interventions deployed to address them, and the invaluable lessons learned.

**Methods:**

Injury characteristics, severity and outcomes of casualties are presented based on the medical records of all casualties arriving to SUMC between October 7th 6:30 a.m. and October 8th 7:00 a.m. Data regarding patient influx, capacity and hospital resource utilization, were collected from the Patient Registration System and other hospital information systems.

**Results:**

During the incident, a total of 673 injured arrived at SUMC within a mere 24-h period, at a peak rate of 83 injured per hour. The mean casualty age was 29.6, with male predominance. Gunshot wounds and shrapnel injuries were the dominant types of injuries. Out of the casualties arrived, about half were hospitalized or transferred to receive definitive care at other hospitals after initial care at SUMC. Mortality rate was low, at 2.9% of those admitted alive.

**Conclusions:**

In this article, we describe the injury characteristics and outcomes of casualties seen at SUMC on October 7th 2023, during one of the largest MCIs in history. We present a detailed overview of the challenges encountered, strategies implemented to address them and lessons learned. These insights hold global relevance, offering actionable guidance for the refinement of future emergency protocols and policies on a global scale.

## Background

Terror attacks are a major cause of Mass Casualty Incidents (MCIs) worldwide. The largest terror attack in terms of causalities per hospital was the attack on the United States embassy in Nairobi, Kenia, in 1998 [1], where Kenyatta National Hospital staff estimated they treated up to 2500 injured on the day of the bombing. Other notable terror attacks are the attack on the World Trade Center on September 11, 2001, in which 2726 people were killed. During this attack, 1,103 patients were seen at the emergency department of St. Vincent Hospital [2, 3]. In addition, 448 casualties reached New York University Downtown Hospital and another 194 reached Bellevue Hospital [4]. In four terror attacks on November 2003 in Istanbul, approximately 700 people were injured, but the case load was divided among many hospitals (On November 15, there were 248 casualties seen in 23 hospitals, while by November 20, 418 casualties were seen in 24 hospitals) [5]. Finally, in the Boston Marathon shootings, 264 patients reached 27 hospitals in Boston [6]. There are also significant examples of major MCIs that are not related to terrorism, such as the Beirut port explosion in 2020 and the Las Vegas shooting in 2017.

On October 7th, 2023, Israel came under the largest terror attack in its history. The attack began at 6:30 a.m. and included launching of more than 3,000 rockets on Israel's civilian areas and an invasion of approximately 3,000 Hamas terrorists [7–10]. More than 1,300 Israelis were killed, mostly civilians, and more than 200 were abducted to Gaza [7–10]. According to Israeli Ministry of Health data, 1,457 people wounded were evacuated to hospitals during the first day of the attack [11], approximately half of them to Soroka University Medical Center (SUMC). This was the largest terror MCI experienced by any Israeli hospital, and one of the largest terror attacks worldwide.

SUMC is located in the city of Beer Sheva and is one of the largest hospitals in Israel. It is part of the Clalit Health System, the largest Health Maintenance Organization (HMO) in Israel. SUMC is the only hospital in the Negev desert in Southern Israel, a tertiary referral and level-1 trauma center with 1175 hospital beds, providing medical services to over one million residents of the Negev. SUMC is the nearest level-1 trauma center to the Gaza strip and the surrounding Israeli kibbutzim and towns. It has extensive experience in emergency scenarios. For example, during the 50 days of Protective Edge military conflict in 2014, the hospital served as the frontline and referral center for those injured, while continuing to cover the regular needs of its catchment population, and functioning under the threat of missile attacks. [12]

## October 7th 2023 MCI uniqueness and challenges

### Casualty volume, influx and flow

During this unprecedented MCI of October 7th 2023, a total of 673 injured individuals arrived at SUMC within the first 24 h. The rate of arrivals was described in a previous manuscript from our medical center, showing that the maximal rate was 83 casualties per hour. [13]

### Long duration, multifocal attack, in a large geographical area

According to estimates, 3,000 terrorists invaded Southern Israel over a range of 841 square km [7–10]. Different foci of the attack included the “Nova” music festival at Kibbutz Re’im, 30 additional civilian locations, including the towns of Sderot and Ofakim, more than 20 kibbutzim, Bedouin villages and 5 cooperative communities, and eight Israel Defense Force (IDF) bases. The different foci were unexpectedly and simultaneously attacked. Efforts to clear terrorist's threat continued more than 72 h, resulting in continuous casualty flow.

### Evacuation challenges

Ongoing armed attacks on the roads leading to hospitals, resulted in injured individuals enduring lengthy delays before evacuation, while the transportation of both staff and casualties to medical facilities faced significant challenges [14].

Casualties arrived by ambulances, helicopters and private vehicles, often without medical care provided prior to arrival at the hospital. Due to the ongoing attack, it was deemed unsafe for ambulances to enter many of the combat areas [14]. Civilians evacuated whoever they could in their private cars. Obviously, these arrivals were unannounced.

Notably, due to the nature of the attack, there was no triage in the field and no direction or prioritizing of patients to specific medical centers, as has been recommended, e.g., following mass shootings in the USA [15].

### Working under fire

In addition to the large number of people evacuated to the hospital at a high rate, SUMC was also under fire. October 7th was the day with the highest number of rockets fired against Israeli population ever, with over 3,000 rockets. The targeting included the areas where most of the hospital staff resides, and the hospital itself, with 18 air-raid sirens sounded at the SUMC perimeter, and 69 alarms in the city of Beer-Sheva [16]. As in previous aerial attacks since 2001, this posed the need to move patients hospitalized in unsheltered departments to more secure locations of the hospital, in addition to halting routine procedures in unsheltered locations [17].

The intricate interplay of these characteristics posed a significant challenge in real time response. The primary aim of this study is to analyze the current incident and outline global lessons for effective MCI management based on this analysis. Through the description of the injury characteristics and outcomes of casualties seen at SUMC on October 7th 2023, we delve into an analysis of one of the largest MCIs in history. We present the challenges encountered, real-time and retrospective assessment of these challenges and strategies implemented to address them. Finally, we present lessons learned that have global applicability and can be integrated into future protocols and policies.

## Methods

### Injury characteristics, severity, and outcomes of casualties

Analysis of injury characteristics and outcomes was based on medical records of all casualties arriving at SUMC on the first 24 h of the attack: between October 7th 6:30 a.m. and October 8th 7:00 a.m.

Casualty data were collected from the electronic and paper medical records. Clinical information was predominantly documented on paper and subsequently digitized into the medical records system. Upon arrival, many patients were initially unidentifiable and were later identified through official documents or by family members. We retrospectively calculated the Injury Severity Score (ISS) to define the severity of trauma. [18, 19]

### Patient influx, capacity, and hospital resource usage

Patient influx information, capacity and hospital resource usage data were collected from the Patient Registration System and other hospital information systems. A manual log of management actions was documented during the event. Data were collected from the manual log, combined with command table protocols and management real-time correspondence.

### Dead on arrival (DOA) definition

Patients were defined as dead on arrival (DOA) according to the proxy definition: unidentifiable Emergency department (ED) heart rate and systolic blood pressure, and Glasgow Coma Scale (GCS) motor component score= 1 [20].

### Pre-hospital data

Patients were evacuated to the hospital by three means of transportation: non-emergency vehicles, ambulances, and helicopters. Information regarding the mode of transportation was not recorded in real-time. Most of the pre-hospital data were also unavailable, in many cases because there was no such care, due to the exceptional security challenges of the incident.

Data collection and analysis was approved by the Institutional Review Board of SUMC, in compliance with the Declaration of Helsinki principles.

## Results

### Baseline casualty characteristics

A total of 673 people were evacuated to the hospital during the first 24 h of the conflict. The mean age was 29.6 ± 14.9 years, with 53 (7.9%) of them 18 years and under, 386 (57.4%) between 19 to 30 years, and 27 (4.0%) above 65 years old. Within the entire cohort, the majority of injured individuals were male (520, 77.3%), and within the civilian injured population, (excluding police officers and soldiers), 291 (69.6%) were males (Table 1). Despite extensive efforts to identify all patients, ultimately, ten individuals (1.4%) remained unidentified as of the writing of the present manuscript, i.e. they were treated and discharged unidentified during the incident.

**Table 1:** Baseline characteristics of the patients

Variable	All patients (N = 673)
*Demographics*	
*Age, years [14 Unknown]*	
Mean ± SD	29.5 ± 14.9
Median (IQR)	24.4 (20.8–35.0)
Males, n (%) *[1 Unknown]*	520 (77.3)
Soldiers, n (%)	223 (33.1)
	[199 males, 24 females]
Policemen, n (%)	27 (4.0)
	[26 males, 1 female]
Civilians, n (%)	418 (62.1)
	*[291 males, 126 females]*
*ED management*	
Treated in trauma crash room, n (%)	122 (18.1)
Chest drain inserted in the ED, n (%)	21 (3.1)
Oxygen support, n (%)	64 (9.5)
Intubated in the ED, n (%)	31 (4.6)
Glasgow Coma Scale < = 13, n (%)	23 (3.4)
*Discharge destination from the ED*	
Admitted, n (%)	239 (35.5)
Transferred to another hospital, n (%)	82 (12.2)
Discharged home, n (%)	336 (49.9)
Dead on arrival	11 (1.6)
Died in ED/immediate operation, n (%)	5 (0.7)
*Emergency department visits after discharge, n (%)*	
48 h	
1 week	12 (1.8)
*Other medical centers*	21 (3.1)
48 h	35 (5.2)
1 week	49 (7.3)
*Injury type*	
Shrapnel injury, n (%)	249 (37.0)
Gunshot injury, n (%)	288 (42.8)
Blast injury, n (%)	39 (5.8)
Anxiety, n (%)	90 (13.4)
Smoke inhalation, n (%)	41 (6.1)
*Location of injury*	
Head, n (%)	59 (8.8)
Ocular trauma, n (%)	21 (3.1)
Ear trauma, n (%)	35 (5.2)
Face, n (%)	53 (7.9)
Neck, n (%)	46 (6.8)
Thorax, n (%)	133 (19.8)
Abdominal and pelvic contents, n (%)	101 (15.0)
Spine, n (%)	24 (3.6)
Upper extremity, n (%)	211 (31.4)
Lower extremity, n (%)	257 (38.2)
External structures (skin, burns), n (%)	51 (7.6)
*Injury score and categorization*	
Injury Severity Score (ISS), Median (IQR)	4 (1–9)
Major trauma (ISS > 15), n (%)	102 (15.2)
*Discharge destination from SUMC hospitalization (n = 239)*	
Died during admission in SUMC, n (%)	5 (2.1)
Transferred to another hospital, n (%)	137 (57.3)
Transferred to rehabilitation, n (%)	10 (4.2)
Discharged home, n (%)	87 (36.4)
*Admitting department in SUMC (n = 239)*	
Orthopedics, n (%)	68 (28.5)
General surgery, n (%)	46 (19.2)
Internal medicine department converted to a surgical department, n (%)	12 (5.0)
Plastic surgery, n (%)	13 (5.4)
Ear nose throat, n (%)	17 (7.1)
Vascular surgery, n (%)	9 (3.8)
Neurosurgery, n (%)	6 (2.5)
Urology, n (%)	8 (3.3)
Intensive care units:	26 (10.9)
General intensive care unit, n (%)	18 (7.5)
Medical intensive care unit, n (%)	8 (3.3)
Cardiothoracic surgery, n (%)	12 (5.0)
Other, n (%)	22 (9.2)

ED- Emergency Department, IQR- Interquartile range, SD- Standard deviation, SUMC – Soroka University Medical Center

### Injury types and severity

The predominant injuries sustained were gunshot wounds (288, 42.8%) and shrapnel injuries (249, 37.0%). After initial triage, 122 (18.1%) patients were evaluated as critically injured and triaged to management in the trauma crash room. A total of 64 (9.5%) patients required oxygen support and 31 (4.6%) required mechanical ventilation (Table 1).

The median ISS was 4 (IQR 1–9), with 102 (15.2%) classified as major trauma (Table 1), including DOA. The most common injuries were to the extremities, with 211 (31.4%) affecting the upper extremities and 257 (38.2%) affecting the lower extremities. The remaining injuries were to the chest in 133 (19.8%), abdominopelvic in 101 (15%) and head injuries in 59 (8.8%) (Table 1).

### Mortality

During hospitalization at SUMC, five patients died, and two more died in other medical centers after being transferred from SUMC (total of seven deaths out of 239 admitted alive, 2.9%, Table 1). In addition, 11 casualties (1.6% of those admitted to the ED) were DOA and 5 (0.7% of those admitted to the ED) died immediately after arrival, at the ED or during emergent operations. Overall, out of 673 patients admitted to the ED, 23 (3.41%) died.

### Casualty influx and flow

As for the rate of arrival at the emergency department, the hours with the highest rate were between 09:00 to 16:00 on Saturday October 7th, 2023. Peak influx of casualties occurred between 12:00–13:00 p.m.

### Capacity and resource utilization

1. Regulation of patient admissions.

Out of all MCI patients, 336 (49.9%) were discharged directly from the ED, while the rest were either hospitalized or transferred to other medical facilities. Hospitalized patients were predominantly admitted to orthopedics, general surgery, or intensive care units (Table 1).

**Fig. 1:**
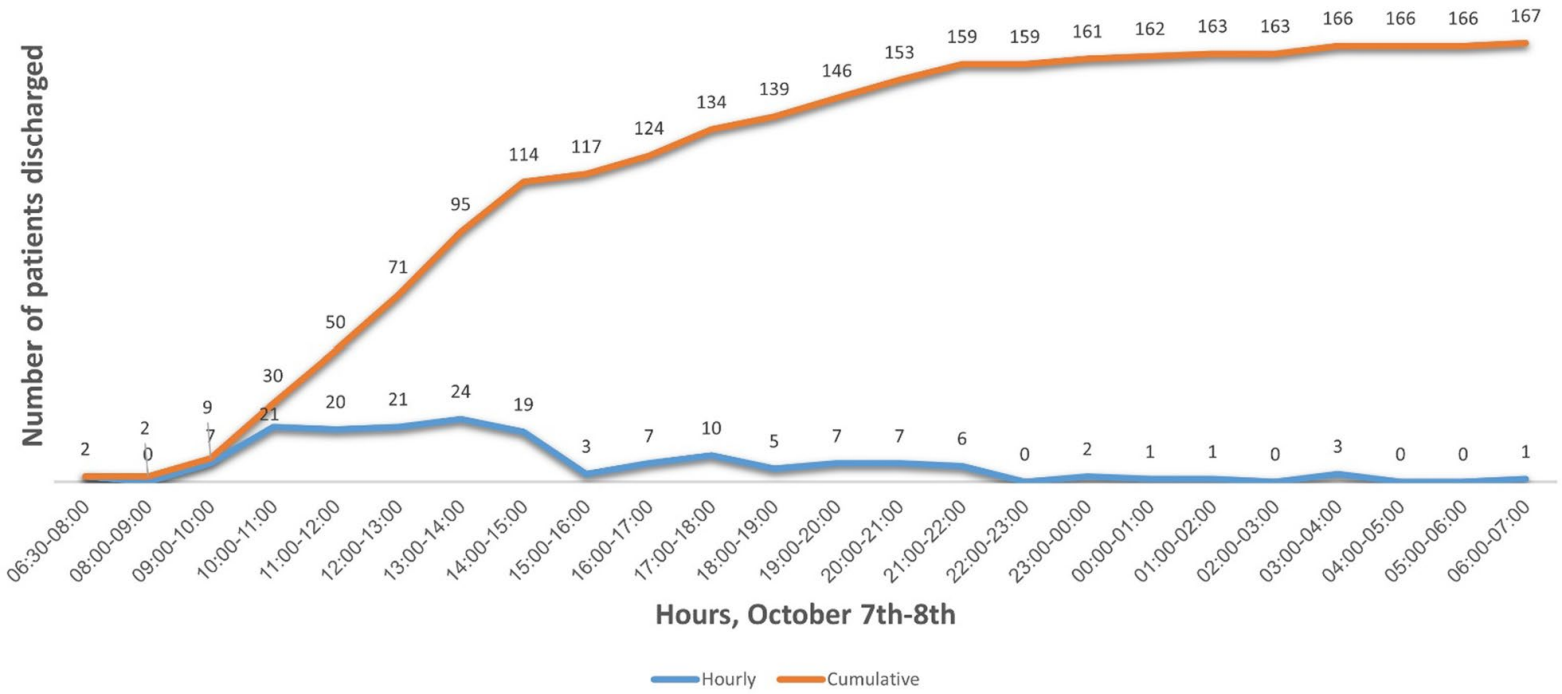
Patients admitted before October 7th, discharged from the hospital during October 7th—all departments (excluding maternity and newborns)

Concomitantly, 167 patients (26.1% of patients hospitalized before the beginning of the MCI, excluding discharges from maternity wards and nursery) were discharged to free hospital beds. (Figure 1).

2. Increasing and regulating hospital resources.

*ICU beds*

Our hospital, with 1175 beds, routinely operates 24 adult ICU beds, with 13 additional designated intensive care beds in neurosurgery and in the cardiothoracic unit. In addition, we have 12 pediatric ICU beds.

*Surgical ward beds*

SUMC routinely operates 62 beds in General surgery, 60 beds in orthopedics and 130 beds in all other surgical sub-specialties.

**Fig. 2:**
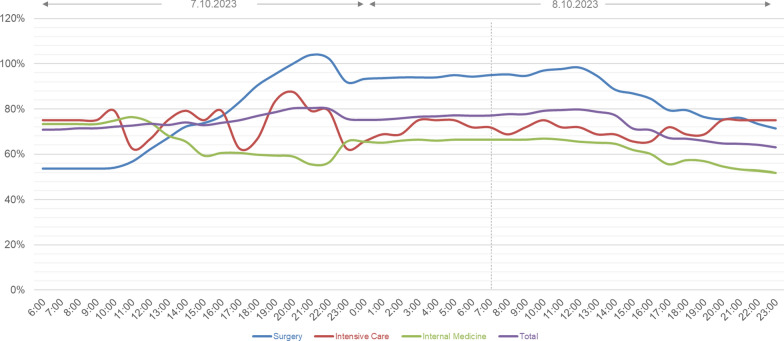
Occupancy of different units and total occupancy at SUMC, during the 7th and the 8.^th^ of October 2023

The occupancy of different units, as well as the total capacity at SUMC during the 7th and the 8th of October 2023, is presented in Figure 2.

*Operating rooms capacity*

SUMC routinely operates 22 operating rooms (ORs). Eight of these are located in unsheltered areas, excluding their use during rocket attacks.

Out of those admitted, 80 patients (33.6%) underwent surgical procedures during the first 24 hours (**Appendix Figure 3**).

*Radiology* *tests*

A total of 153 patients underwent 168 computed tomography (CT) scans, with total body CT (trauma protocol) being the most common test (91 patients, 13.5% of the total). Additionally, 332 patients underwent 703 x-ray examinations within that 24-hour period (**Appendix Figure 4**).

*Blood products*

Throughout these 24 h, a total of 254 units of packed red blood cells and 41 units of whole blood were administered, with 134 and 37, respectively, given in the trauma crash room. Typical blood product usage, under routine conditions, is usually 30–60 units of packed red cells per day, with rare use of whole blood. The standard inventory of packed red cells is 150–200 units.

*Human resource recruitment and management*

Of more than 5000 employees, 1700 were present at the hospital during the first 24 h of the MCI. Three-hundred and sixty physicians out of 900 were present at various departments during the event, 95% more than routine normal Saturdays. Out of those present physicians: 32.9% were surgeons, 12.9% anesthesiologists, 7% emergency medicine physicians, 2.8% intensive care physicians, 11.8% internal medicine physicians, 8.1% pediatricians, 2.8% psychiatrists, 2.8% radiologists and 4.8% gynecologists.

## Discussion

### From October 7th, 2023 MCI, to strategies and global lessons

We outlined the injury patterns and outcomes of casualties treated at SUMC during the extreme MCI of October 7th, 2023. Based on SUMC's response to the emerging extreme challenges of the event, we analyze strategies implemented, and suggest global lessons that, in our view, can be learned and used for future emergency preparedness (Table 2).

**Table 2:** Key challenges and lessons for dealing with a mass-casualty incident

Challenge	Lesson
*Before the event*	
Clear protocols	Have clear protocols for emergency preparedness including the roles of various staff members in an emergency
Frequent drills	Drill MCI frequently so that the staff is familiar with their roles in an emergency. Make sure to include all sectors
Consider the best alternative for documentation	Decide whether to use paper records or computer-based records in an MCI and use it as part of the drill. Make sure records are easy to use
Define the structure of the incident command system	Consider working with a central command desk and emergency complexes dealing with specific issues such as clinical decision, logistics, manpower
*During the event*	
Early declaration of the MCI	Do not wait for external activation; gather information from the outside as soon as possible
Announcing an MCI	Err on the side of over-stretching the system, rather than be surprised that you are under-staffed for the number of casualties
Capacity management	Discharge all patients that do not have to stay at the hospital; transfer all patients who can be moved to medical / surgical floor out of the ICU
	Consider secondary evacuation of patients who have been stabilized but require further surgical procedures to other hospitals as early as needed and possible
Expect the unexpected	Consider the possibility of a multi-focal event, extensive geographical extent and / or prolonged duration and non-conventional modes of evacuation of casualties (e.g. private vehicles)
Role of emergency medicine and intensive care physicians	Consider managing the various sites in the emergency department by emergency medicine and / or intensive care specialists, saving the trauma experts for clinical evaluation of individual challenging patients and emergency surgical procedures
Resource management	Make sure you have enough operating rooms, or use additional sites (e.g. OB/GYN, day-care surgery room) when relevant
	Consider using additional CT scanners such as the CT component of a PET-CT or the simulation CT for radiotherapy. Map hardware and software capabilities in advance
	Bring additional equipment to the trauma room to increase the number of patients who can be treated simultaneously
Communication interruptions	Consider the possibility of local or extensive disruption of cellular communication. Use alternatives such as satellite phones
Logistics	Make sure you have enough equipment (e.g. surgical supplies) and medications and look for quick solutions for replenishing the stock
Handling corpses	Locate an alternative site for corpses if the mortuary is overloaded
*Following the event*	
Improve documentation	Complete all missing documentation. If documentation was done in paper records, scan or type (preferred) them into the electronic medical record
Debriefing	Debrief as early as possible and reflect on what went well and what could have been improved
Improve your readiness	Based on the debriefing, consider changes to emergency protocols as needed
Share the knowledge	Share the knowledge with other hospitals in the country and worldwide, to improve emergency preparedness in the future

CT- Computerized Tomography, ICU- Intensive-Care Unit, MCI- Mass- Casualty Incident, OB/GYN- Obstetrics& Gynecology, PET- Positron Emission Tomography

A Mass Casualty Incident is an incident in which the number of patients exceeds the resources normally available locally. [21, 22]. The October 7th 2023 MCI is by far the largest MCI in the history of Israel and one of the largest worldwide. In comparison, a paper summarizing 20 MCIs in central Israel [23] reported a mean number of casualties of 30.2 ± 19.5, (range 9–66). This paper also summarized global MCIs due to terrorism and the median number of casualties per hospital was 136 [23]. Similarly, between 2000 and 2018 there were 373 MCIs with civilian society targets in Europe, with 15,066 casualties (the median number of casualties was 11 (range7-26) for MCIs due to explosives, and 9 (range 6–22) for MCIs due to firearm attacks). [24]

During the October 7th 2023 MCI, a total of 673 injured arrived at SUMC in 24 h, at a peak rate of 83 injured per hour. Similar to other terror attacks, the injured population was relatively young (mean age 29.6 years, compared to 30–39 years in previously described terror attacks, [2, 25, 26] and most of the casualties (77.3%) were male (previous described range from 66 to 77%) [2, 25, 26]. The percentage of patients with major trauma was relatively high (15.2%) compared to some previous incidents [2, 4] and admission rates (35.5%) were within the previous range of 6% to 64% [2–5, 25, 26]. The rate of patients requiring ICU admission (10.9%) was also similar (5.2–38%) [25, 26]. Mortality (2.9%) was similar to the range of previously reported statistics, despite the increased severity of wounds (2.8–37.5%). [2, 4, 25, 26]

### Meeting extreme challenges

#### Unique event characteristics

This event was unique due to a combination of the extreme rate of casualty influx, a long duration and multifocal nature of the attack, and challenges of pre-hospital treatment and evacuation. Each one of these manifestations can be challenging by itself and requires preparedness.

1. Casualty influx and flow:

One of the critical factors in trauma care of patients in MCIs is the rate of casualty arrival [27]. The maximal rate of arrival was 83 casualties per hour [13]. The flow of patients can be even higher: in the Boston Marathon bombing, all 264 patients injured arrived at several hospitals within 18 minutes, while during the World Trade Center attack in September 11, 2001, 150 to 175 patients per hour were seen at the beginning of the event. [2–4]

2. Long duration, multifocal attack on a large geographical area:

The event described was multifocal and prolonged. The attack took place in different foci simultaneously, including the “Nova” music festival, and 30 additional locations. The area was cleared of all terrorists only after more than 72 hours. In comparison, out of 373 MCIs in Europe between the years 2000-2018, 86% were unifocal and of a short period. Among the multifocal ones, the maximum number of sites recorded in a single incident was 8 (Russia in 2005 and Paris in 2015) [24]. This is important because emergency preparedness should take into account the ability to manage the incident at multiple simultaneous foci in a coordinated manner. A multifocal MCI makes it difficult to manage resources and communication between the different sites. [24]

3. Evacuation challenges:

The Pre-hospital response time and modes of evacuation are of great importance in MCIs. It has been previously shown that response time of emergency medical services (EMS) can be delayed dramatically as an impact of MCIs in the same area [28]. In the described incident, the ongoing shooting during the evacuation rendered it impossible for ambulances to access many areas [14], prolonging the critical time from injury to arrival at SUMC and compelling civilians to undertake the evacuation of casualties in their own vehicles, without medical treatment in the field besides the placement of tourniquets. Unlike standard operations where EMS communicate with the emergency department *en-route* to facilitate trauma team preparation, these arrivals occurred without any forewarning. Similarly, due to the extreme circumstances, some helicopters landed and delivered casualties unexpectedly, without advance notification.

## Early recognition and announcement of MCI

In a scenario like the present one, it is of utmost importance to recognize the potential severity of the situation and identify it as an MCI as early as possible. Every minute in mobilization counts. Indeed, the attack started at 6:30, with hundreds of rockets fired all over southern and central Israel and multiple attacks across the Gaza strip border. Based on information received informally from the frontline, it was understood as early as 7:40 a.m. that a large-scale attack was going on. The hospital emergency preparedness and leadership teams convened at 7:45 a.m. and MCI was announced at 8:00 a.m., after only 12 casualties had arrived to the hospital. Since the night shift was ending, the staff was asked to stay and provide care. When an MCI was announced, many physicians, nurses, and other hospital staff arrived from their homes (although it was a Saturday and a holiday).

### Early and independent casualty classification and mobilization

It was essential to treat casualties by severity. Therefore, per MCI protocol, we operated several sites according to the severity of injury, with critically injured casualties treated in the trauma room. In addition, there were designated sites for moderately and severely injured, mildly injured, and anxiety-related injuries. All routine activity of the ED was shifted to one site within the ED. Each site had a senior physician and a nurse in charge, pre-assigned in advance according to hospital protocols. One of the sites was the triage post, managed by an emergency medicine specialist and a senior nurse. This team saw all incoming casualties and assigned them to the various sites.

Regarding casualty flow, we embrace the “unidirectional flow” scheme adopted also by other hospitals [23]. The main concept emphasized by this plan is that each patient goes through an individualized process, in which patients move from one station to the next according to their needs, and do not return to a previous station. For instance, for patients treated in the trauma room, this means they move on to imaging, surgery or admission and can move forward but not return to a previous site, particularly not to the trauma crash room.

### Role of various specialty care teams in event management

We agree with other authors [4, 29, 30] regarding the importance of having a senior physician experienced in trauma present in triage, a physician experienced in trauma coordinating the overall surgical response, and having attending level physicians supervising key areas. Nevertheless, we emphasize the pivotal role of intensive care, anesthesiology, and emergency medicine specialists, as an active and major part in the current incident management and implementation [31]. We offer to consider site management in relevant sites by intensive care and/or emergency medicine specialists, saving the trauma experts for individual challenging cases or surgical interventions in need.

### Capacity and resource utilization

Regarding capacity challenges, we had to increase the availability of resources, such as trauma unit beds, ICU beds, CT scanners and operating rooms. In order to balance the supply and demand of resources, actions were taken to minimize demand as well.

1. Regulation of patient admissions:

Of injured inpatients, 137 were transferred to other hospitals within the first 24 hours. Another 44 were transferred to other hospitals within the first 48 hours.

In addition, similar to other major MCIs [4, 26], patients in the internal medicine and surgical wards whose condition allowed it, were discharged home to make room for casualties from the incident.

This process of hospitalization regulation required complex and fast team cooperation, including medical, nursing and social work staff, in addition to hospital- community cooperation, and national resources regulation by the Ministry of Health.

2. Cancelling elective activity:

Given that it was a Saturday, when there are no planned elective surgical cases, there was no need to cancel them; however, as a lesson for other MCIs which might happen at any day of the week, such surgical procedures are often cancelled immediately. [4, 26]

3. Increasing and regulating hospital resources:

Similar to other authors [31–33], we stress the importance of rapid identification of resources and potential problem areas, and matching resources to needs.

*ICU Beds*

In addition to 24 adult ICU beds, 13 designated intensive care beds in neurosurgery and cardiothoracic unit, and 12 pediatric ICU beds, our emergency protocols include the operation of an 8-bed unit within less than 24 hours, utilizing equipment purchased during the COVID-19 pandemic and nursing staff recruited from other medical centers in Israel. This was indeed functional and operational within 12 hours.

*Surgical ward beds*

After rapid and efficient patient mobilization from the internal medicine departments, we converted a 38-bed internal medicine unit into a surgical unit, staffed with physicians from surgery and internal medicine and a nursing staff based on internal medicine.

*Trauma room capacity*

As for infrastructure in the trauma room, we brought in additional equipment and converted our 6-bed trauma room into a 12-bed functional trauma unit.

*Operating rooms capacity*

Since 8 of our 22 operating rooms are not bomb-sheltered, we utilized 3 of the rooms in our obstetrics and gynecology division for abdominal surgeries.

*Radiology tests*

CT scans were performed for 23% of patients. This created a significant load on our single CT scanner located in a sheltered location, within the ED. As a solution, we operated two additional scanners, one of which was a CT simulator used for radiotherapy planning and the other – the CT component of our positron emission tomography–computed tomography (PET-CT). These are located in sheltered areas and could be utilized during the ongoing rocket attack. [13]

*Logistic supply*

Surgical supply, medications, blood products and additional equipment were continuously assessed, reported on an hourly basis, and supplied from central resources accordingly, during the first 24 hours and the following days.

4. Human resource recruitment and management

Despite the potential danger, and the fact it was a Saturday and a holiday, about 1700 of more than 5000 employees answered our call and came to work that day. In addition, we requested and received help from other medical centers: 28 nurses and 18 physicians (general surgeons, anesthesiologists and orthopedic surgeons) arrived to help by the afternoon hours. [13]

## Communication interruptions

A potential challenge which could occur in an MCI is communication interruptions. For example, during the World Trade Center attack, hospitals had to use “messengers” between parts of the hospital for information exchange, due to collapse of the cellular network. Volunteers, such as medical students, were used for such a challenge [4]. We also experienced a disruption of communication during the incident, as the cellular network at our operating rooms floor was temporarily unavailable during the event. Following the event, we purchased radio and satellite communication devices for communication with agencies outside the hospital.

### Global lessons for emergency preparedness

#### Expect the unexpected

Information from the field was haphazard. We had no real-time information regarding the number of casualties, severity of injuries, projected timelines, mode of transportation, etc. This improved as the day evolved. According to informal information in combination with emergency preparedness team experience, MCI was declared as early as 8:00.

#### Emergency practice strengths: strengths of routine are strengths in emergency

One of the major strengths of our hospital stems from frequent MCI drills, both by the Ministry of Health and independently by our emergency preparedness team. In addition, SUMC has extensive experience in actual MCIs, both in times of war [12] and in times of peace. The significance of well-defined protocols cannot be overstated. Previous reports [24] stress the importance of emergency preparedness. Drills should include all personnel in the hospital, as nonmedical personnel including administrative support and housekeeping staff carry out functions that are integral to efficient operations of the department. [24]

#### Redefining the hospital incident command system: emergency complexes

Lessons from the COVID-19 pandemic were used in this event [34, 35]. These include the need for senior leadership to be present at the frontline, and having frequent assessments of challenges and resources, every few hours, in a methodology of hospital Incident Command System. Working in team complexes, each team dealing and revising a specific field: clinical issues, logistics, human resources, media relations etc. on a daily basis. We started working with this method from the second day following the MCI. Each complex summarized actions and recommendations and brought them daily to a central table of command [36–39]. This method is implemented worldwide and was increasingly used during the COVID19 pandemic. [34, 35]

#### Manual MCI record disadvantages

During MCIs, due to time constraints, primarily the need for rapid evaluation and decisions, documentation is performed on paper charts. Pre-organized paper files, which in Israel are nationally designed, were found to be cumbersome and were not uniformly used as intended. We found that these files need to be redesigned to be more intuitive, and that staff needs to be trained more frequently in their correct use. We have also designated a staff member to ascertain correct use in early stages of future MCIs. Some limitations of using paper records during an MCI are that paper-based systems are easily marred, destroyed, or lost. Staff are used to electronic systems and may be unfamiliar with paper systems [6]. We believe that designers of electronic systems should be challenged to create an electronic system to document clinical data in real time during MCIs. Despite the use of paper charts, electronic records have become crucial for patients in all scenarios including MCIs. This is required to ascertain continuity of care, as providers have become dependent on electronic medical records with discrete data recorded. This is also important for various benefits that terror victims are entitled to. We led a process of information digitation into the electronic medical record system (beyond scanning) after the event, and learned the importance of integrating the process as part of future protocols.

#### Monitoring and strengthening staff resilience

The continuing firing of rockets and terror attacks led to anxiety of the staff for their loved ones at home, and the need to cope under fire. Nevertheless, staff commitment was so high that people worked unabatingly for long hours and did not hesitate to stay at the hospital during the attack. Staff resilience was a critical component of recruitment, teamwork and collaboration, and is a key component in the hospital competency.

The hospital resilience team arrived as early as 8:00 a.m. at the beginning of the event and was present at the ED, to give emotional support to the staff and learn about staff experiences. Soon after the event, this team operated a mapping process of high-risk exposure groups. 1600 workers were recognized as exposed to possible hazardous experiences, from which a total of 150 workers were eventually recognized as high-risk for developing moral and emotional difficulties and received intensive support and suitable care.

#### Navigating the Threat of Chemical, Biological and radiological (CBRN) Terrorism

While this MCI involved conventional weapons, a terror attack of this magnitude can involve chemical, biological and radiological (CBRN) agents, as well. CBRN weapons had been rarely used (0.2% of terror attacks 1970–2019 globally, yet they had extremely high potential to inflict mass casualties with mean non-fatal injury rates of 50% and 29% for chemical and biological weapons, respectively. [40] In a study of violent non-state actor CBRN events, there were a total of 565 events in 1990 to 2020. Of these, 67% involved chemical agents, 13% involved biological agents, 7% radiological and 2% nuclear, while 10% included a combination of several types of CBRN agents. Of these, 22 attacks occurred in Israel, with 12 fatalities and 180 injured [41]. Some notable terror attacks involving chemicals included two sarin attacks by the Aum Shinrikyo in the city of Matsumoto in 1994 (n = 500 injuries), and in the city of Tokyo in 1995 (N = 5,500 injuries). Furthermore, 1500 people were injured by 60 rockets holding mustard gas in Kirkuk, Iraq in 2016. In other attacks, chlorine gas, poison and ‘other unknown’ gases were listed. There was one attack with a biological agent resulting in a large number of casualties: the deliberate contamination of salad bars at ten local restaurants with Salmonella typhimurium in Oregon, United States, in 1984, causing 751 injuries [40]. The modern approach to toxicological MCIs involves early decontamination in the fields, even before specialist response is operated. Dry decontamination may have several advantages over aqueous (shower based) decontamination [42]. A few recommendations for dealing with a toxicological MCI can be listed. These include (1) ensuring access to laboratory analysis as soon as possible; (2) strict adherence to personal protective equipment requirements; (3) effective communication to break through bureaucracy, enabling timely decisions across multiple agencies; (4) paying attention to wellbeing and psychological support for all staff and finally (5) collaboration and teamwork. [43]

#### The importance of post MCI investigation and debriefing

Finally, it is important to debrief, learn from successes and identify gaps, and disseminate knowledge [4] by presenting data to others. This is one of the incentives for writing the present paper, as well as a previous short description. [13]

A summary of the challenges and our identified generalizable lessons are detailed in Table 2.

### Study strengths and limitations

Our study analyzes the real-time handling and outcomes of the largest MCI in Israeli history. The large casualty volume allows comparison of the results to previous large MCIs and has potential of generalizability for future preparedness. The extreme circumstances of several casualty surges upon 24 consecutive hours, under rocket fire and continuous undefined terror threat on access roads are unique in comparison to any other extreme scenario-based drills. Nevertheless, data are limited to a single center. Due to proximity to frontline events, the moderately- and severely-injured might have been selectively referred and treated at SUMC, in a way that influenced injury severity. Data with regard to the mode of transportation to the medical center, as well as pre-hospital treatment information, lacks in part.

## Conclusions

The mass casualty incident at SUMC during October 7th, 2023, can serve as a pivotal example of an extreme emergency scenario that was never experienced before. This article analyses the real-time management mechanisms during the incident, identifying areas of success and opportunities for improvement. This event highlights both the strengths and weaknesses of present emergency response protocols. Furthermore, it points out several key components for evolving protocols, to ensure readiness for extreme future events and ultimately safeguard the well- being of both patients and healthcare providers. Under complex extreme circumstances, 673 casualties received emergency care, with outcomes that align with existing literature. The key element in managing MCIs is continuous preparedness. We highlight critical lessons to be learned from this historic horrific event.

## Figures and Tables

**Fig. 3:**
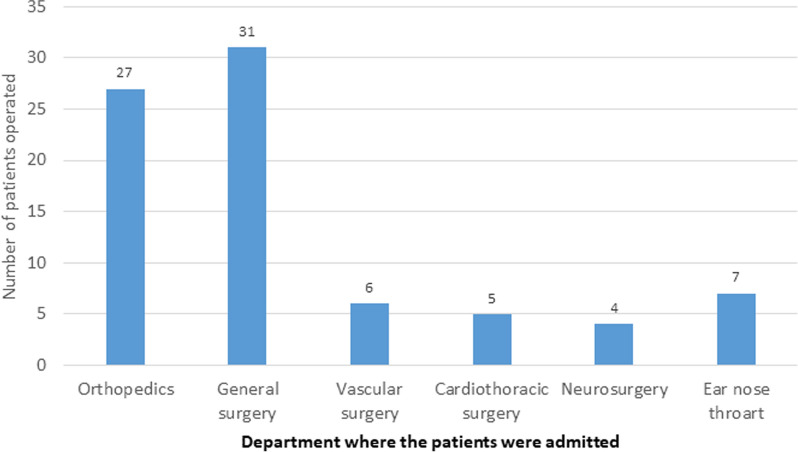
Surgeries during the first 24 h (total number of patients operated – 80)

**Fig. 4:**
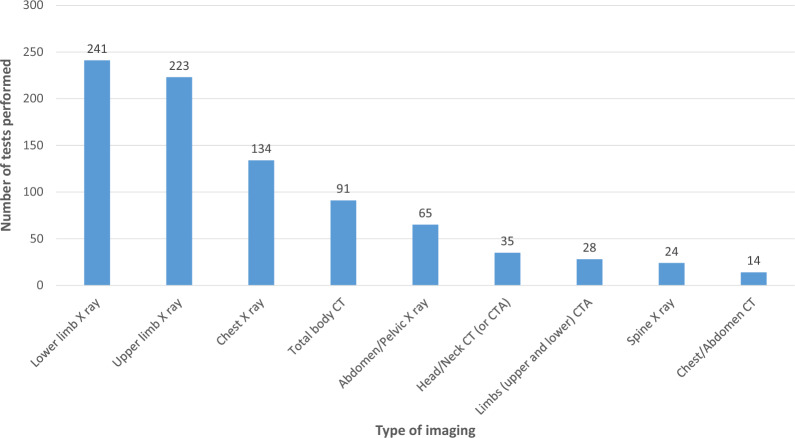
Imaging during the first 24 h. (703 X rays for 332 casualties, 168 CT scans for 153 casualties). CT – computed tomography, CTA – computed tomography angiography

## Data Availability

Data generated and analyzed during this study are included in this published article and its supplementary information files.

## Appendix

See Figs. 3 and 4.

## Abbreviations

SUMC: Soroka university medical center
MCI: Mass casualty incident
DOA: Dead on arrival
IDF: Israeli defense forces
